# Silver Nanoparticles Based Ink with Moderate Sintering in Flexible and Printed Electronics

**DOI:** 10.3390/ijms20092124

**Published:** 2019-04-29

**Authors:** Lixin Mo, Zhenxin Guo, Li Yang, Qingqing Zhang, Yi Fang, Zhiqing Xin, Zheng Chen, Kun Hu, Lu Han, Luhai Li

**Affiliations:** 1 Beijing Engineering Research Center of Printed Electronics, Beijing Institute of Graphic Communication, Beijing 102600, China; beiyinguozhenxin@163.com (Z.G.); zqq15201169516@163.com (Q.Z.); fangyi@bigc.edu.cn (Y.F.); zhiqingxin@bigc.edu.cn (Z.X.); hukun@bigc.edu.cn (K.H.); hanlu@bigc.edu.cn (L.H.); 2 Research Institutes of Sweden (RISE), RISE Bioeconomy, Drottning Kristinas väg 61, 11428 Stockholm, Sweden; li.yang@ri.se; 3 Shine Optoelectronics (Kunshan) Co., Ltd., Shenzhou Industrial Park, No. 33 Yuanfeng Rd, Kunshan 215300, China; zchen2015@163.com

**Keywords:** silver nanoparticles, flexible and printed electronics, moderate sintering, protective agent, substrate modification, photonic sintering, transparent conductive film, biosensor

## Abstract

Printed electronics on flexible substrates has attracted tremendous research interest research thanks its low cost, large area production capability and environmentally friendly advantages. Optimal characteristics of silver nanoparticles (Ag NPs) based inks are crucial for ink rheology, printing, post-print treatment, and performance of the printed electronics devices. In this review, the methods and mechanisms for obtaining Ag NPs based inks that are highly conductive under moderate sintering conditions are summarized. These characteristics are particularly important when printed on temperature sensitive substrates that cannot withstand sintering of high temperature. Strategies to tailor the protective agents capping on the surface of Ag NPs, in order to optimize the sizes and shapes of Ag NPs as well as to modify the substrate surface, are presented. Different (emerging) sintering technologies are also discussed, including photonic sintering, electrical sintering, plasma sintering, microwave sintering, etc. Finally, applications of the Ag NPs based ink in transparent conductive film (TCF), thin film transistor (TFT), biosensor, radio frequency identification (RFID) antenna, stretchable electronics and their perspectives on flexible and printed electronics are presented.

## 1. Introduction

Over the past few decades, silver nanoparticles (Ag NPs) have made a substantial impact on various fields, such as biomedical [1,2,3], optoelectronics [4,5], catalysis [6,7,8,9], imaging [10,11,12], etc., due to their superior physical, chemical and biological characteristics compared to their macroscale counterparts. For instance, Ag NPs have made great progresses in the development of novel antimicrobial agents [13,14,15,16], drug-delivery formulations [17,18,19], detection and diagnosis platforms [20,21,22], performance-enhanced biomaterial and medical devices [23,24], etc. In the emerging and fast growing multidisciplinary research field, flexible and printed electronics (FPE), Ag NPs have also been a key component of conductive ink [25,26,27]. FPE refers to the application of printing technologies for the fabrication of electronic circuits and devices on flexible substrates [28,29]. It differs from the traditional manufacturing technologies of electronic devices, e.g., photolithography, vacuum deposition and electroless plating process. The traditional technologies involve multiple steps, require high cost equipment and production environment (clean room), and the use of environmentally undesirable chemicals, which result usually in the formation of large amounts of waste. In contrast, FPE may be viewed as an additive manufacture method that brings about the possibility of preparing relatively high-resolution devices in a much simpler, faster and more cost-effective way.

Like other emerging science and technologies, advances in materials [30,31,32,33,34,35] have been a major driving force for FPE, including printable organic and inorganic materials: conductive, semi-conductive and insulative. Among the conductive materials, Ag NPs hold a unique position when making high performance conductive ink because of their high electric conductivity and good oxidation resistance. For Ag NPs based printed electronics, there are two major factors that dominate the conductivity of the printed device, e.g., packability of Ag NPs and sintering. The morphology and size distribution of Ag NPs are responsible for packability. A good packability means a dense Ag NPs based film structure, which is essential for good conductivity. After Ag NPs based conductive ink was printed on the substrate, the sintering process is often needed to remove or decompose the protective agents from the surfaces of Ag NPs, enabling direct physical contacts between Ag NPs, and to establish a dense and conductive network throughout the printed feature. As the devices are usually printed on heat sensitive flexible substrates, it is crucial to keep the sintering in a moderate condition. Thus, obtaining Ag NPs based ink, which only requires for moderate sintering and high conductivity, is of the utmost important for the development of FPE.

In this review, recent developments in Ag NPs based conductive inks with moderate sintering and their applications in the FPE are summarized, with particular emphasis on the methods and mechanisms to achieve highly conductive Ag NPs based ink under moderate sintering. The review describes the relevant strategies in Section 2, including tailoring the protective agents capping on the surfaces of Ag NPs, optimizing the sizes and shapes of the Ag NPs, and substrates modification. Some emerging sintering technologies, e.g., infra-red sintering, intense pulsed light sintering, laser sintering, electrical sintering, plasma sintering and microwave sintering, are also included. Applications of the Ag NPs based ink for FPE devices are presented in Section 3, including the transparent conductive film, thin film transistor, biosensor, stretchable electronics and radio frequency identification antenna. Finally, we conclude this review with a summary and discussions on the perspectives and challenges of the Ag NPs based ink and the related sintering techniques in FPE areas in Section 4.

## 2. Strategies of Achieving Highly Conductive Ag NPs Based Ink under Moderate Sintering

For Ag NPs based ink, sintering means that the Ag NPs begin to make physical contact with each other and form a continuous percolating network in the printed pattern. To achieve a high conductivity, further sintering is required to transform the initially very small contact areas into thicker necks and, eventually, to a dense layer. In the initial stage of sintering, the driving forces are mainly surface energy reduction due to the Ag NPs’ large surface-to-volume ratio, a process known as Ostwald ripening [35]. Ostwald ripening triggers surface and grain boundary diffusion within the coalesced Ag NPs. Grain boundary diffusion allows for neck formation and neck radii increase, which is diminished by the energy required for grain boundary creation. As the sintering develops into a deep level, the relative density of the printed Ag NPs based film increase and the electric conductivity increase too. In this section, we focus the attention on the strategies of obtaining highly conductive Ag NPs based ink under moderate sintering and their mechanisms. The key influential factors related to the moderate sintering of Ag NPs based ink, such as protective agents, Ag NPs size and shapes, substrate modification as well as the emerging selective sintering techniques, are discussed in the following.

### 2.1. Protective Agents

Protective agents are commonly used to improve the stability of the metallic nanoparticles suspension. It is well known that the protective agents could be adsorbed onto the surface of the nanoparticles thus controlling their nucleation and growth rates as well as preventing agglomeration and sedimentation of the prepared nanoparticles [36,37,38]. Meanwhile, the adsorbed protective agents, even though as thin as a few nanometers or only in a mono molecular layer, are found to prevent electrons from moving between the metallic nanoparticles and decrease the conductivity of the printed film [39,40]. Thus, post-treatment is usually employed to reduce the protective agents covering and to sinter the metallic nanoparticles, both resulting in improved conductivity. Therefore, a better understanding of the sintering process as well as the effects of the protective agents on the conductivity of the printed Ag NPs based pattern is needed. Usually, two kinds of protective agents are commonly used in Ag NPs based inks: first, the polymers bearing carboxylate, amino or hydroxyl functional groups, such as poly(acrylic acid) (PAA) [41,42,43,44], poly(vinyl pyrolidone) (PVP) [45,46,47,48,49] and poly(vinyl alcohol) (PVA) [50,51]; second, the small molecular compounds with a long alkyl chain and polar head, such as alkanethiols [52,53,54], alkylamines [53,55,56] and carboxylic acids [57,58]. Through investigating the behavior of protective agent in sintering, some efforts have been made to improve the conductivity of the Ag NPs based ink under moderate sintering.

**Figure 1:**
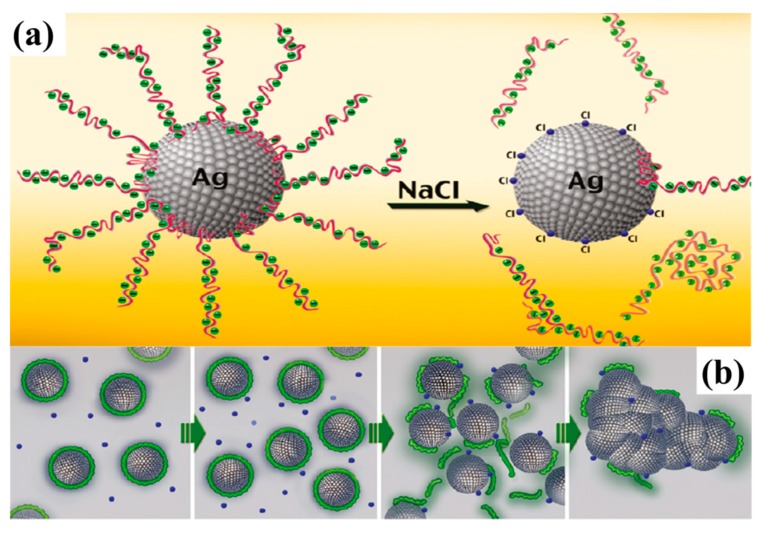
(**a**) schematic illustration of the Ag NPs before (left) and after (right) the addition of NaCl, and (**b**) schematic illustration of the protective agents detachment, which leads to the Ag NPs sintering (the green lines represent the polymeric stabilizer; the blue spheres represent the sintering agent). Reproduced with permission from [42]; Copyright 2011 American Chemical Society.

**Figure 2:**
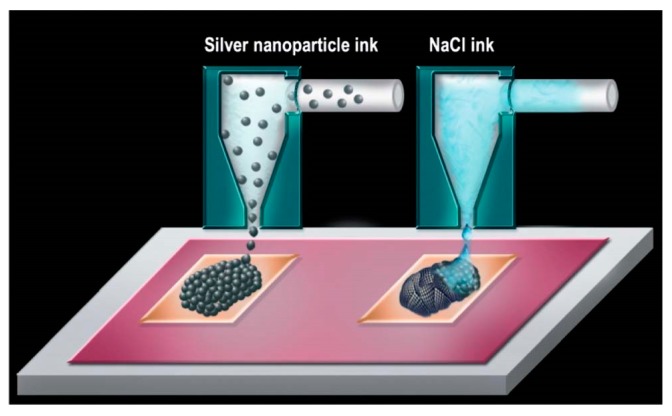
Scheme of the double printing process. First, a pattern of Ag NPs based ink is printed, followed by printing a salt solution on top of the silver pattern. Reproduced with permission from [59]; Copyright 2012 Royal Society of Chemistry.

Magdassi et al. [41], Grouchko et al. [42] and Tang et al. [46] realized room temperature sintering of the Ag NPs capped polymer protective agents by adding the destabilizing agents, oppositely charged Cl^-^ containing electrolyte, into the ink to promote the Ag NPs aggregation and coalescence in the drying processes. The optimized electric conductivities achieved were 20%, 41% and 40%, respectively, of that of bulk silver. The destabilizing agents, which contain Cl^-^ ions, cause detachment of the anchoring groups of the protective agents from the surface of Ag NPs and thus enable their sintering (Figure 1). Further study showed that this sintering is dependent on coalescence and Ostwald ripening spontaneous behaviors of Ag NPs after they have been destabilized. In addition, these two behaviors could be extremely affected by the size of the Ag NPs [46]. On this basis, Layani et al. [59] reported a rapid and simple process to obtain high conductive printed patterns, above 30% of bulk silver, by sequential printing of the Ag NPs based ink and solutions of electrolyte such as NaCl and MgCl_2_ (Figure 2).

The influence and behavior of small molecules protective agents on the conductivity and sintering of the Ag NPs based ink were also investigated. Previously, we prepared Ag NPs, with dodecylamine (DDA) and dodecanethiol (DDT) as the protective agent, and studied the effect of protective agents on the properties of the Ag NPs based film in the post-treatment [53]. The results showed that the molecular structure of the DDA and DDT as well as the bonding strength between the protective agents and the Ag NPs surface affect the conductivity, sintering temperature and morphology of the Ag NPs based film significantly. The bonding energy of Ag-S being higher than that of Ag-N and a higher alkyl chain ordering of capping DDT molecules lead to a stronger interaction between the alkyl chains than that of capping DDA molecules. Thus, Ag-DDA film requires a lower treatment temperature to convert it into conductive than that of Ag-DDT film. The results showed that the printed Ag-DDA NPs based film even could transfer from insulative into conductive with an electric resistivity as low as 15.1 μΩ·cm after air storage at room temperature for less than seven days. In addition, the electric resistivity of the Ag-DDA NPs based ink after 60 min heat-treatment at 140 °C reached 2.9 μΩ·cm, which is 1.8 times the bulk Ag resistivity. Jung et al. [60] achieved low temperature sintering and highly conductive Ag NPs based ink by ligand exchange and ligand reduction using an acetic acid (AA) immersion treatment. The original surface capping agent of oleylamine (OA) was replaced by AA through the ligand exchange, simultaneously resulting in the capping ligand weight reduction by 10 wt.%. The ligand exchange was explained by the difference in adsorption energy of the two ligands, as estimated by density functional theory (DFT) calculation. The relative energy difference between the state of OA being adsorbed and the state of AA being adsorbed is approximately −1.98 ev. Thus, AA adsorption is energetically much more favorable than the OA adsorption. Both the reduced ligand weight and relatively lower bonding energy between Ag NPs and ligand contributed to the lower sintering temperature of the Ag NPs based ink compared to its counterpart before ligand exchange.

### 2.2. Ag NPs Sizes and Shapes

**Figure 3:**
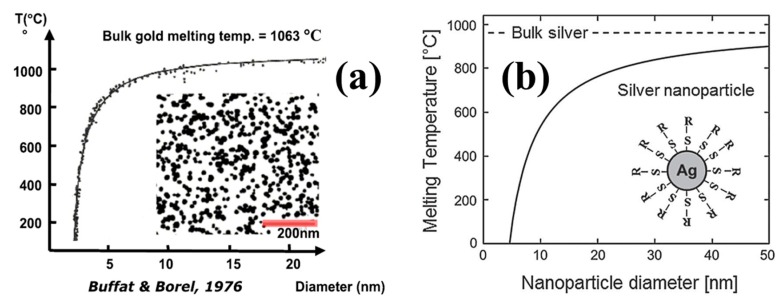
Experimental and theoretical values of the melting point temperature of (**a**) gold particles. Reproduced with permission from [61]. Copyright 1986 American Physical Society; (**b**) silver particles as the function of decreasing particle size; reproduced with permission from [62]; Copyright 2006 Springer.

It is well known that nanomaterials usually exhibit novel specific properties that may be significantly different from that of bulk materials in mechanical, optical, electrical, thermal and magnetic properties. For instance, according to the phenomenological model and the experimental observations presented by Buffat and coworker (Figure 3a) [61], the melting temperature of gold particles significantly drops when the diameter is smaller than 5–7 nm. This size dependent melting temperature decrease is also observed and investigated in Ag NPs [62]. The Ag NPs approximately 2 nm show melting behavior at significantly low temperatures (≈150 °C) compared to the melting temperature of bulk Ag (960 °C), as illustrated in Figure 3b. This huge melting temperature depression is not only very interesting from a fundamental research perspective, but indicates that the atomic diffusion becomes very active in nanoparticles near the surface which is very important for the flexible and printed electronics applications requiring low temperature processing. On the other hand, the shape and size distribution of the nano-Ag fillers in conductive ink could affect the packing density, filler interconnect and morphology of the printed film during post treatment process, which have a significant impact on the conductivity and sintering of the Ag NPs based ink [63].

Balantrapu et al. [64] and Ding et al. [65] studied the relationship between the size distribution and electrical properties of the printed Ag NPs based film. The results showed that the electric resistivity and sintering of the printed pattern are highly dependent on the Ag NPs size distribution. The Ag NPs based ink with bimodal distribution or relatively broad size distribution is more favorable to form extensive conductive 3D network in the printed pattern during sintering by forming a large number of contact points in different sized Ag NPs. In addition, the voids caused by volumetric shrinkage of the relatively large Ag NPs during sintering could be filled with relatively small Ag NPs, resulting in a compact morphology and high conductivity of the printed film. The optimal electrical resistivity values of ~6.7 μΩ·cm and ~3.83 μΩ·cm were achieved by Balantrapu et al. and Ding et al. at 200 °C and 160 °C, respectively. Seo et al. [66] focused their research on the effects of both the Ag NPs size and the type of protective agents on the conductivity and morphology of the Ag NPs based film during the sintering process. It was found that the size of the Ag NPs was the main factor influencing the initial decrease in the resistivity because of the neck formation between Ag NPs and the type of protective agents was the most important factor for determining the final resistivity of the conductive films due to interconnections of the Ag NPs via extended neck formation. The lowest resistivity (2.2 μΩ·cm) was obtained for the film that was prepared using 3.4 nm Ag NPs, hexylamine as a stabilizer, and sintered at 220 °C. Han et al. [67], Yang et al. [68] and Lee et al. [69] investigated the shape influence on the electrical property of the nano-Ag based film by using the Ag NPs (spherical shape), nanorods, nanoplates and their mixtures as the conductive fillers. It was found that, when combining the Ag NPs with Ag nanoplates or nanorods at a certain ratio as the conductive filler, the different shapes of nano-Ag mixture based ink demonstrate a higher conductivity at a relatively low temperature compared to that of single Ag NPs based ink. The conductive mechanism research shows that the small sized Ag NPs provide sufficient energy to motivate the grain and lattice transport to facilitate strong bonding and the large sized Ag nanoplates or nanorods stack densely to reduce the porous space in the pattern. Specifically, Han et al. obtained the resistivity of 10.3 μΩ·cm at 100 °C for 30 min which was only 6.5 times of the bulk Ag by mixing Ag NPs and nanoplates with the weight ratio of 1:1.

### 2.3. Substrate Facilitated Sintering

In the above sections, we have discussed that the sintering and electrical property of the Ag NPs based ink could be tailored by controlling the property of protective agents and optimizing the shape and size distribution of Ag NPs. In this section, we pay attention to another key component of the flexible and printed electronics: the substrate. It is well known that the requirements when printing for electronics are totally different from those for printing graphic arts. Graphic printing needs images or text with a good visual impression, whereas electronic applications require continuous and homogeneous patterns with restrictions on the layer thickness, roughness, and print resolution. Therefore, the substrates, whether plastics or papers, must be able to offer some or most of the following properties: thermal stability, dimensional stability, barrier properties, solvent resistance, low coefficient of thermal expansion, a smooth surface and optical clarity for display purposes. MacDonald et al. [70] reported the issues associated with the selection of a plastic film with the required property set for development and the leading candidate materials for plastic-based flexible electronics. In addition, Tobjörk et al. [71] reviewed recent progress in the development of electronic devices on paper substrates.

A recent research provides an extremely interesting approach, where substrate modification leads to the spontaneous coalescence and sintering of Ag NPs at a relatively low temperature. This substrate facilitating sintering of Ag NPs is attributed to two aspects’ reasons, which are mainly related to the superficial physical and chemical properties of the substrates, respectively. The superficial physical properties include the surface roughness, solvent wettability, solvent absorption rate and mechanical stability, etc. The chemical modification of the substrates is intended to provide chemical removal of the protective agents from the surface of the Ag NPs, which is in accordance with Refs. [70,71,72,73] in Section 2.1. While the main distinguishing factor of the chemical related substrate facilitated sintering compared to the methods mentioned in Refs. [70,71,72,73] of Section 2.1 is that the sintering agent is added in the paper coating during manufacturing and do not need any post treatment of the printed Ag NPs pattern. This is significant for the large-scale production and high speed roll to roll printed electronics.

Lee et al. [72] characterized the commercial available photo-papers with respect to their superficial physical and chemical properties to obtain highly conductive Ag NPs based printed patterns at a relatively low sintering temperature. The results showed that chloride ions on the paper’s surface when they are under a certain value could activate the decomposition of polymer protective agent and sintering between the Ag NPs. On the other hand, the surface roughness and pore size of the paper were inversely related to the conductivity of the Ag NPs pattern.

**Figure 4:**
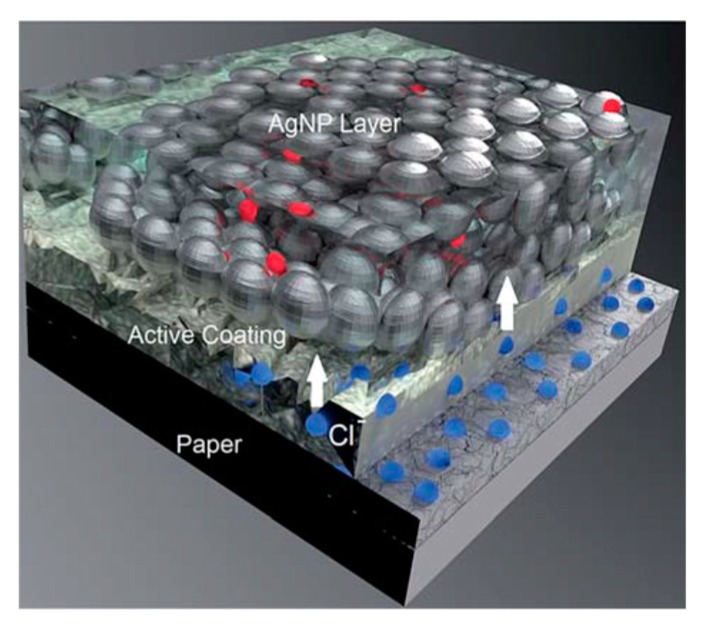
Schematic image showing the principle of the active papers. A small amount of chloride is contained in the coating as a sintering agent. During the deposition of the Ag NPs dispersion and absorption of the carrier fluid, Cl ions migrate into the Ag NPs film and react with the Ag NPs matrix to assist the sintering. Reproduced with permission from [73]; Copyright 2015 Royal Society of Chemistry.

Öhlund et al. [73] incorporated the sintering agent of chloride as an ingredient of the mesoporous paper coating to achieve chemical sintering and investigated the effect of the variations in the pore size of paper coating and precoating type on the sintering of Ag NPs. Figure 4 shows that the Cl^-^ migrate into the Ag NPs film when Ag NPs deposit in the printing process and react with the Ag NPs matrix to assist the low temperature sintering. Meanwhile, the sintering is impaired by increasing the pore size of the paper coating, but greatly enhanced by using a porous CaCO_3_ precoating.

Allen et al. [74] and Andersson et al. [75] also found that, by choosing the type of ink receptive coating, it is possible to manufacture printed Ag NPs based pattern without the need for, or at least to reduce the need for, post print sintering. Allen et al. [74] demonstrated that the room temperature sintering of Ag NPs could be achieved on the substrates with the ink receptive coating that contains silanol groups. The silanol groups could dissolve the protective agent of PVP on the Ag NPs surface by providing enhanced water absorption in the substrate coating layer as well as providing strong binding sites so that it is energetically favorable to detach the protective agent from the Ag NPs. Andersson et al. [75] observed an extreme difference in electric resistivity for tracks printed on paper substrates with aluminum oxide based coatings compared to silica based coatings. Nge et al. [76] paid attention to obtain the superficial nanostructured paper and studied its influence on the electrical property of the inkjet printed Ag NPs patterns. They introduced a direct sheet casting method to prepare cellulose nanofibers (CNF) based paper, with unique surface features including a nanoporous network structure and low surface roughness. The CNF based paper shows a shorter sintering time at a low temperature and a less pronounced coffee ring effect compared to the commonly used paper and plastic because of the permeation of the ink vehicles through the nanopores and absorption along the nanofibrils that compete with the initial spreading and the final evaporation process.

### 2.4. Photonic Sintering Method

**Figure 5:**
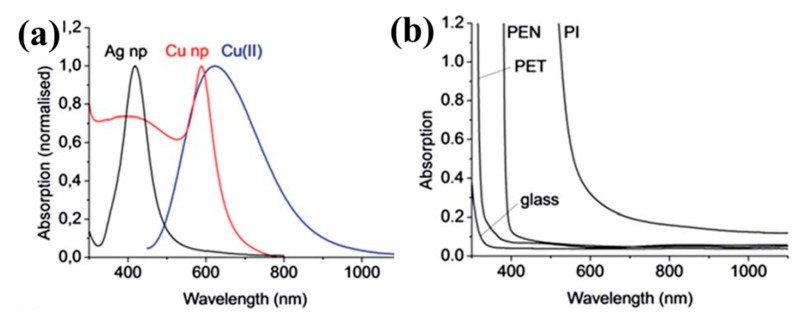
(**a**) UV-Vis absorption spectra of commonly used metallic NPs dispersion of conductive inks and (**b**) substrates for printed electronic applications. Reproduced with permission from [77]; Copyright 2014 Royal Society of Chemistry.

Recently, various emerging sintering techniques have been used to obtain highly conductive printed patterns based on Ag NPs ink under moderate condition. In this section, the photonic sintering, which is the most popular method in this related field, is presented. The sintering of metallic NPs based inks via electromagnetic (EM) irradiation ranging between the ultra-violet (UV) and infra-red (IR) is called photonic sintering. Frequently reported bands are in the infra-red (IR), ultra-violet (UV) and visible region, which is called intense pulsed light (IPL) or photonic flash sintering. Since the absorption of metallic NPs based inks (plasmon resonance) is in the visible region (Figure 5a), UV irradiation (ranging from 100 to 400 nm) is not suitable for the selective heating of these materials but mainly applied to metal organic compounds (MOD) inks, which is not in the discussion scope of this review. In addition, a special form of irradiation is laser sintering, where the emission of the laser can be tuned in a narrow wavelength window or even a single wavelength to match the absorption spectrum of the respective ink formulation. Rather than heating the entire system indiscriminately, photonic sintering enables targeting specific components selectively, leaving the substrate that tends to absorb only in the UV range (Figure 5b, the polyimide substrate is the exception because of its brown color) unaffected.

#### 2.4.1. Infra-Red (IR) Sintering

**Figure 6:**
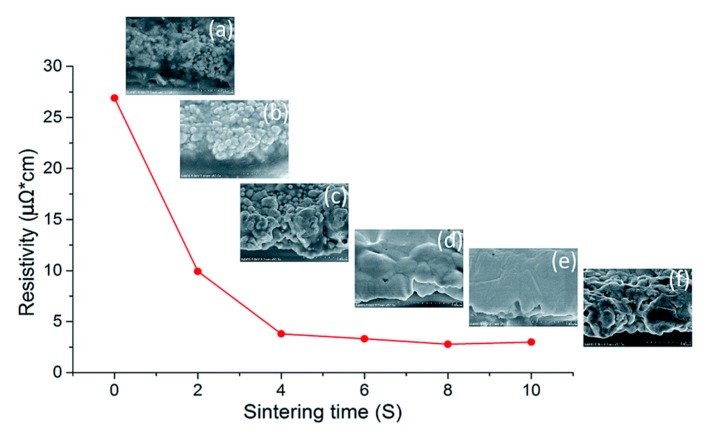
The resistivity of Ag NPs film sintered by NIR with power of 360 kW∙m2 over 10 s and SEM images of the sintered film at (**a**) 0 s; (**b**) 2 s; (**c**) 4 s; (**d**) 6 s; (**e**) 8 s,;and (**f**) 10 s. Reproduced with permission from [80]; Copyright 2018 Royal Society of Chemistry.

IR technology using irradiation in the range of the NIR to MIR region (700 to 15,000 nm) facilitates the contact-less and selective drying and sintering of printed metallic NPs based layers within a very short time. Denneulin et al. [78] used an IR lamp operating at wavelengths of 8 to 15 μm to sinter the inkjet printed pattern of Ag NPs. A similar level of electric resistance was obtained by IR sintering within a relatively short time of 3 min compared to that by conventional heating at 200 °C for 5 min. while the high wavelength of the using IR also caused a fast temperature increasing of the substrate to 180 °C–210 °C, which limits its application on the temperature sensitive substrate. A more selective approach of IR sintering was performed by Cherrington et al. [79], who used irradiation in the near-IR (NIR) region to sinter the slot-die coated Ag NPs pattern on Polyethylene terephthalate (PET) substrate within 2 s yielding a conductivity of about 16% of bulk Ag. Irradiation in the NIR is shown to be less absorbed by the used PET, enabling a selective sintering of the metal ink without substrate deformation. The NIR irradiation was also used by Tobjörk et al. [52] and Gu et al. [80] to sinter printed Ag NPs inks on paper and plastic substrates. An optimal sintering result can be achieved by carefully adjusting settings like power output, distance between lamp and sample and treatment time. The resistivity of 2.78 μΩ·cm was achieved after only 8 s exposure to NIR irradiation with no damage to the substrate, which was only 1.7 fold higher than that of bulk Ag. Figure 6 shows the electrical resistivity and morphology evolution of the printed Ag NPs based film during sintering process [80].

**Figure 7:**
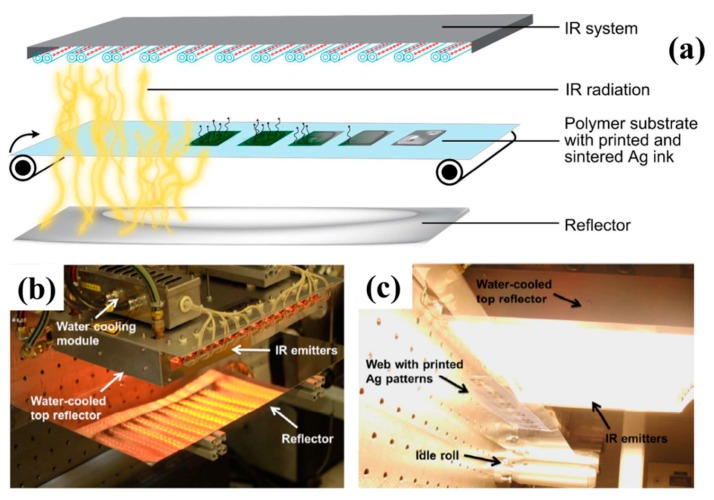
(**a**) scheme of the experimental setup of roll to roll (R2R) IR drying and sintering of inkjet-printed Ag NPs layers on Polyethylene naphthalate (PEN) substrates. The R2R sintering instrument in (**b**) top view and (**c**) from below with activated IR radiation. Reproduced with permission from [81]; Copyright 2015 Royal Society of Chemistry.

Sowade et al. [81] reported a roll to roll (R2R) NIR drying and sintering process for inkjet printed Ag NPs layers on Polyethylene naphthalate (PEN) substrate (Figure 7). Relevant process conditions, e.g., intensity of IR radiation, duration of exposure, velocity of moving substrate, usage of IR reflectors, the distance between IR emitters and printed Ag NPs layers, were varied to evaluate the effects on the morphology and conductivity of sintered Ag NPs layer. The optimized electric conductivity up to 15% of Ag bulk was achieved at high web velocities up to 1 m/s with an exposure time of less than 0.5 s. Basically, IR sintering is a very fast (in the order of seconds) method to sinter Ag NPs based inks to obtain conductivity values in the range of 10%–35% of the Ag bulk. Considering heat dissipation from the printed Ag NPs coating into the substrate happened also very fast, the sintering parameters should be carefully optimized and the paper substrate with high diffuse reflectance, relatively high thermal stability and low thermal conductivity is especially suitable.

#### 2.4.2. Intense Pulsed Light (IPL) Sintering

**Figure 8:**
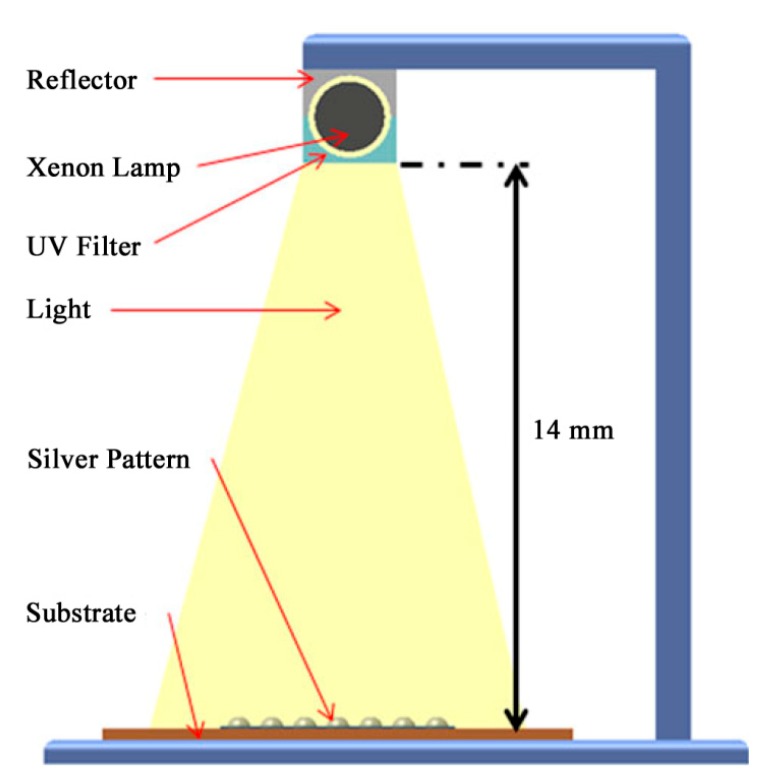
Schematic of intense pulsed light (IPL) sintering for Ag NPs based film using xenon flash irradiation. Reproduced with permission from [82]; Copyright 2011 Springer.

Intense pulsed light (IPL) or photonic flash sintering is essentially a thermal technique which employs the heat generated by the absorption of visible light in the target materials to achieve the necessary temperature increase. In contrast to conventional thermal sintering, where the sample is exposed continuously to a high temperature, IPL irradiates the sample with multiple short flashes, each with a pulse length in the range of a few micro-to milliseconds. The most commonly used light source for IPL sintering is a xenon stroboscope lamp, which emits radiation in the range between roughly 200 and 1200 nm, encompassing the entire visible spectrum. Figure 8 gives the schematic of IPL sintering of Ag NPs based film [82].

Although, in most cases, the IPL was used to sinter Cu NPs based ink because of its superiority in the reduction of the oxide layer on the surface of Cu NPs, a number of reports concerning Ag NPs have appeared about the influence of various IPL parameters on sintering time, final conductivity, film morphology and substrate damage. Chung et al. [83] obtained the optimal IPL sintering conditions for the gravure offset printed Ag NPs film on PET substrate by in situ monitoring of the IPL sintering process. The optimized IPL process reduced the sheet resistance of Ag NPs based film to below that of thermally sintering without damaging the PET substrate or allowing interfacial delamination between the Ag NPs film and PET. Kang et al. [82], Abbel et al. [84], Lee et al. [85] and Sarkar et al. [86] investigated the effect of the IPL parameters such as flashing frequency, intensity, pulse duration and number on the electrical property and morphology of the Ag NPs based film. The results showed that variation of the IPL sintering parameters offers a wide range of conditions for process optimization. In addition, the ink composition and type of substrate also have a decisive influence on the IPL sintering of Ag NPs based ink. According to the investigation of Lee et al. [85], the protective agent and organic additives play a critical role in the microstructure formation inside IPL sintered film, which affects the final electric resistivity. The vaporization induced from the thermal decomposition of the protective agent and organic additives could result in film swelling during the re-melting stage of the surface Ag NPs layer. Weise et al. [87] presented and analyzed the application of IPL sintering on inkjet printed Ag NPs based patterns on various flexible substrates, like PEN, PET, Polyimide (PI) and paper. A high dependency of the electrical and structural properties of the printed Ag NPs layer on the substrate was observed. This observation was explained as resulting from the different surface roughness, solvent absorbing rate and thermal conductivity of the substrates.

#### 2.4.3. Laser Sintering

**Figure 9:**
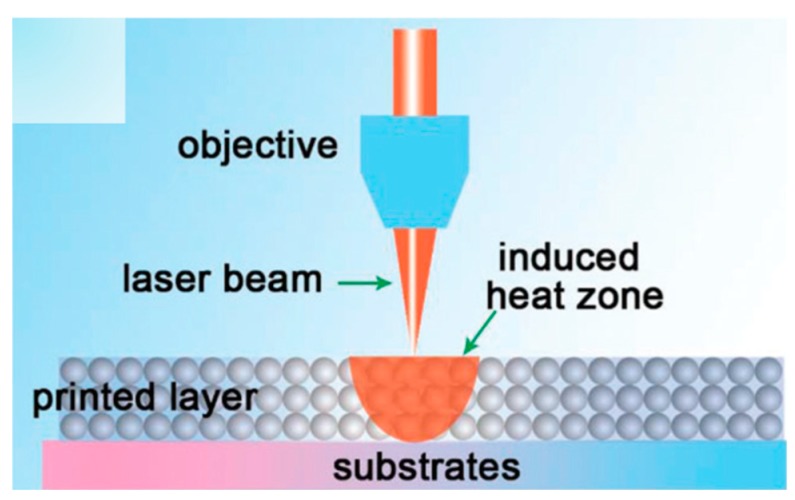
Scheme of laser sintering principle: a focused beam locally heats the printed Ag NPs layer. Reproduced with permission from [32]; Copyright 2017 Royal Society of Chemistry.

Laser sintering has shown great promises to achieve high-quality sintering locally through controlling the heat penetration to preserve the substrates’ integrity. The printed Ag NPs based layer absorbs the laser irradiation in the affected area followed by heating up and sintering the Ag NPs due to the photothermal effect shown in Figure 9. The generated temperature inside of the Ag NPs based layer has to be controlled and kept as low as possible to avoid heat dissipation into the substrate material. Thus, a careful adaption of sintering parameters like power output, writing velocity, wavelength and operation mode (continuous wave or pulsed) should be carried out allowing a reduction of the processing temperatures on the substrate. Balliu et al. [88] investigated laser sintering of inkjet printed Ag NPs inks on papers. High conductivity of 1.63 × 107 s∙m^−1^, nearly 26% of the bulk Ag, was achieved where a special care was taken in sintering parameters to prevent the substrates from damage by intense laser light. Yeo et al. [89] sintered the R2R printed Ag NPs layer by laser to a conductivity up to 20% of bulk Ag on PET substrates. Bolduc et al. [90] indicated that controlling the incident laser pulse’s energy distribution in the time-domain was paramount to optimizing sintering process in Ag NPs based ink. A multi-step microsecond-pulsed laser process and a time-domain pulse-shaping modulation sintering caused a uniform and high conductive printed Ag trace on polymer substrates.

**Figure 10:**
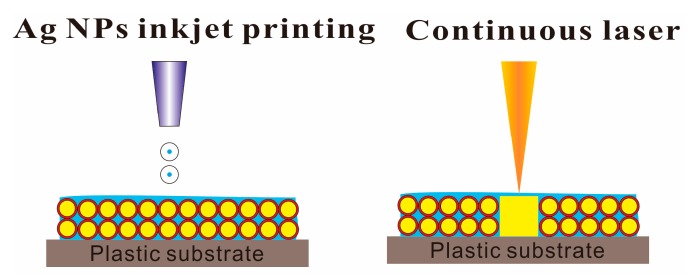
Selective laser sintering process of inkjet printed metallic NPs on a polymer substrate. The circles represent metallic NPs with protective agents and the square block indicates a conductor pattern of sintered metallic NPs. Unsintered NPs are simply washed away in an organic solvent.

**Figure 11:**
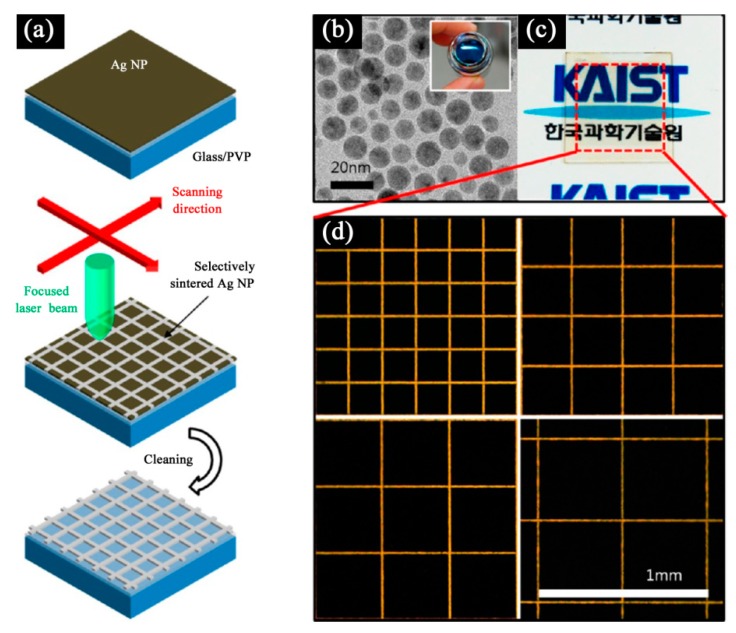
(**a**) schematic diagram of selective laser sintering of Ag NPs for the fabrication of a transparent conductor; (**b**) TEM: Transmission electron microscope (TEM) image of synthesized Ag NPs (inset: optical photograph of Ag NP ink); (**c**) photograph of a transparent conductor on a glass substrate (metallic grid in the red-boxed region); (**d**) optical stereoscope images of square-metallic grids at different grid sizes (200 to 500 μm, increment 100 μm). Reproduced with permission from [92]; Copyright 2013 American Chemical Society.

In the other hand, the spot size of the laser and its heat affected zone is far more smaller than the minimum trace of printing technologies, which makes laser sintering a suitable tool for high resolution and lithography free manufacturing [91]. Figure 10 shows the selective laser sintering process of metallic NPs based ink. Hong et al. [92] fabricated a metallic grid transparent conductor on PET and glass substrates using selective laser sintering of Ag NPs based ink (Figure 11). Such the transparent conductor with high transmittance (85%) and low sheet resistance (30 Ω/sq) could be produced at a large scale without any vacuum or high temperature environment.

### 2.5. Other Emerging Sintering Methods

**Figure 12:**
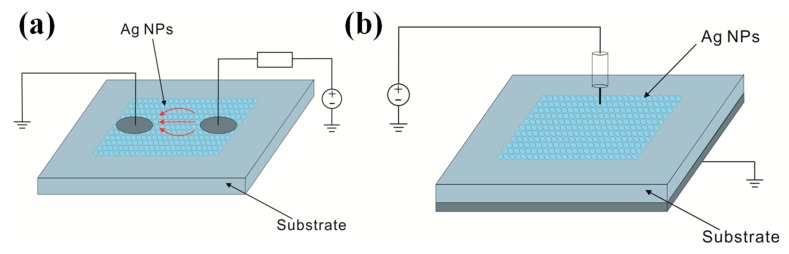
(**a**) schematic illustration of a contact-mode Direct current (DC) electrical sintering setup and (**b**) contactless Alternating current (AC) sintering between a probe above the NPs layer and a ground plate beneath the printing substrate.

Electrical sintering describes the application of a current to printed Ag NPs based inks causing local heating within the ink, which is due to its highly resistive nature before sintering. This process occurs on a timescale of a few milliseconds to seconds and is called rapid electrical sintering (RES). RES is demonstrated on printed Ag NPs structures by applying direct current (DC) voltage as well as via a near-field coupled alternating current (AC) electric field [93]. Figure 12a illustrates a sintering setup, where sintering electrodes are in contact with the Ag NPs layer. When a voltage U is coupled between the sintering electrodes, a non-zero current flow (indicated by arrows in Figure 12a) causes local heating in the layer. This initiates the sintering process and the structure undergoes a rapid transition in conductivity. The series resistor Rs limits the maximum current once the structure is sintered. Contact-mode electrical sintering has been applied using DC voltage. However, the requirement of directly contacting the printed pattern during sintering demonstrates an obstacle in large quantity fabrication. Therefore, contactless electrical sintering using AC current was developed. This is accomplished by applying sintering electrodes above the sample, which couple to the printed layer (Figure 12b). Allen et al. [94] obtained excellent conductivity up to 60% of bulk Ag in very short time of 2 μs using DC current with the power density of at least 100 nW/μm^3^.

Plasma sintering is usually performed by exposure of printed patterns to low pressure Ar plasma. During plasma exposure of the Ag NPs based ink, the plasma inherent active species decompose the protective agents on the surface of Ag NPs due to chain scission, which results in the sintering of the Ag NPs. The sintering process shows a clear evolution starting from the top layer into the bulk. Reinhold et al. [95] used a low pressure argon plasma in order to sinter Ag NP inks on glass, PC and PET to a conductivity of up to 30% of bulk Ag. Recently, Wolf et al. [96] reported that low pressure Ar plasma sintering resulted in the conductivity of printed patterns equal to 11% of bulk silver after only 1 min of exposure, and 40% of bulk silver after 60 min of exposure, while the processing temperature was below 70 °C. To avoid the need for sophisticated equipment for low pressure plasma sintering, Ar plasma sintering at atmospheric pressure and room temperature was developed by Wünscher et al. [97,98]. With this technique or combining with a mild heating of the substrate less than 110 °C, relatively high conductivity of the printed Ag NPs based trace was obtained in a short sintering time without substrate damage. This approach enables sintering of patterns printed onto plastic substrates and can be utilized in R2R processes. Ma et al. [99] sintered the Ag NPs film on glass substrate by applying the Ar plasma and studied the effects of plasma conditions on the morphology, composition and electrical property of the sintered Ag NPs film. The optimized resistivity of the sintered Ag NPs film was about five times higher than bulk Ag.

Microwave radiation also can be used as an alternative and selective sintering technique [100]. Typically, Ag NPs based film have a penetration depth of 1–2 μm at a microwave frequency of 2.45 GHz. While since the Ag NPs is a good thermal conductor, the printed pattern will be heated uniformly by thermal conductance enabling the microwave radiation to be applied to sintering patterns with thickness exceeding the penetration depth. In contrast to the relatively strong microwave absorption by the Ag NPs, the polarization of dipoles in thermoplastic polymers below Tg is limited, which makes the polymer substrate transparent to microwave radiation. Perelaer and his colleges focused their research on the microwave sintering of the Ag NPs based ink for many years. Their research results showed that the exposure of inkjet printed Ag NPs to microwaves decreased the sintering time by a factor of 20 with the conductivity value of 5% compared to the bulk Ag [100]. Further decreasing the sintering time to only a few seconds with the conductivity up to 10% to 34% of the bulk Ag have been achieved by placing conductive antennae structure around the Ag NPs based pattern [101]. This process can be implemented into R2R production. Meanwhile, combining microwave and other sintering techniques have been proved to be an effective way to improve the sintering performance. Combining photonic and microwave flash treatments enabled obtaining 40% of bulk silver conductivity in less than 15 s [102]. Even higher conductivity, 60% of bulk Ag, was obtained in less than 10 min by combining low-pressure Ar plasma and microwave sintering of printed Ag NPs on PEN foil without damage of the polymeric substrate [43].

## 3. Applications of the Ag NPs Based Ink

In this section, we will discuss several applications of Ag NPs based ink and their perspectives in FPE. This will include fabrication and properties of transparent conductive film (TCF), which are essential features nowadays for many optoelectronic devices, printed thin film transistor (TFT), biosensor, radio frequency identification (RFID) antenna as well as emerging stretchable and wearable electronics.

### 3.1. Transparent Conductive Films

Indium tin oxide (ITO) with both excellent transparence and conductivity has been the most widely used transparent conductive film (TCF) in decades. However, an ITO film also has a number of unavoidable disadvantages and weaknesses, such as the relatively high cost and poor flexibility. As the development of the large area and flexible devices such as solar cells, touch panel, light-emitting device and display, extensive efforts have been made to obtain alternatives to ITO [103,104,105,106,107,108]. Among alternative materials and approaches, patterned Ag NPs grids are a promising candidate for high performance TCF. For instance, we prepared a high-performance ITO-free TCF by combining high-resolution flexography printed Ag NPs grids with a carbon nanotubes (CNTs) coating [45]. The Ag NPs grids/CNTs hybrid TCF with a 20 μm grid width at an interval of 400 μm exhibits excellent overall performances, with a typical sheet resistance of 14.8 Ω/sq and 82.6% light transmittance at room temperature as well as good mechanical flexibility. Magdassi et al. [103] produced TCF by inkjet printing of diluted Ag NPs based inks to form overlapping metallic rings, forming in spontaneous self-assembly of Ag NPs during solvent evaporation. The resulting array Ag NPs based ring with rims <10 μm in width and <300 nm in height has a transparency of 95% and sheet resistance of 4 Ω/sq. Ahn et al. [104] produced the TCF with high transparency of 94.1% by direct writing of concentrated Ag NPs based ink. Deganello et al. [105] obtained TCF with a transparency of 81.4% and sheet resistance of 1.26 Ω/sq by patterned micro-scale Ag NPs based grids using roll-to-roll flexographic printing. Kahng et al. [106] obtained highly conductive flexible TCF with a sheet resistance of 12 Ω/sq and 73% transparency at 550 nm by combining ink-jet printed Ag NPs grids with graphene film. In addition, Jeong et al. [107] obtained an Ag NPs grid/ITO hybrid TCF by inkjet printing. The hybrid TCF has a sandwich structure with the Ag NPs grids in the middle of two ITO layers, showing a sheet resistance of 2.86 Ω/sq and transparency of 74.06%.

Ag NPs grids based TCF exhibits excellent performance in terms of optical transparency, electrical conductance, and mechanical flexibility. Thus, they have found applications in many optoelectronic devices such as displays, touch screens, organic light emitting diodes (OLEDs) and solar cells. Many proof-of-concept devices such as solar cells and OLEDs with incorporated TCF have been demonstrated. Li et al. [108] reported using Ag NPs grids based TCF to fabricate ITO-free flexible organic solar cell. The Ag NPs grids’ TCF has very fine honeycomb structure with the width of around 3 μm and the diagonal length of 130 μm, showing low sheet resistance less than 5 Ω/sq and high transparency of 85%. A layer of highly conductive PEDOT:PSS(3,4-ethylenedioxythiophene): poly(styrenesulfonate) on the top of Ag NPs grids is added to increase the charge collection efficiency. Flexible organic solar cell using this TCF as electrode, and P3HT:PCBM as the photoactive layer, achieved a PCE of 1.36%. When using high performance conjugated polymer, PTB7, as the donor, the highest power conversion efficiency of 5.85% was achieved for a large area flexible polymer solar cell [109]. Cai et al. [110] reported a novel electrochromo-supercapacitor based on an Ag NPs/PEDOT:PSS hybrid transparent electrode. The bifunctional device performs as a regular energy storage device and simultaneously monitors the level of stored energy with rapid and reversible color variation, even in high current charge/discharge conditions.

### 3.2. Thin Film Transistor

**Figure 13:**
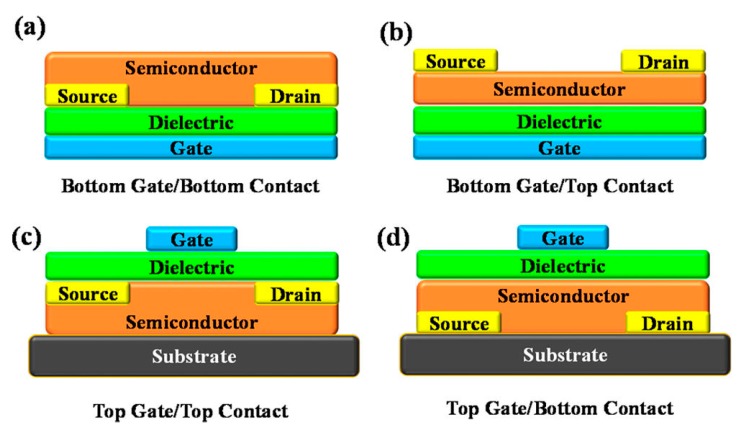
Schematic diagram of four types of TFTs (TFTs: Thin film transistors); (**a**) bottom gate/bottom contact structure; (**b**) bottom gate/top contact structure; (**c**) top gate/top contact structure; (**d**) top gate/bottom contact structure.

Thin film transistor (TFT) is an electronic device widely used in applications including display back plane, sensor and logic circuit, etc [111,112,113]. In addition, it is considered as a model device to investigate the intrinsic physics of semiconductor film and metallic electrode contact, which also are significant for other devices, such as photovoltaic cell and organic light emitting diode. TFTs are constructed with four parts at least: an active semiconductor film usually named as channel layer, a dielectric layer used as gate insulator, a gate electrode and a couple of source/drain electrodes. Figure 13 shows a schematic of four types of TFTs [114].

The printing fabrication of electrodes avoids the conventional vacuum deposition of metallic films, and is expected for full-printed devices. More importantly, the etching inevitable during the photolithograph process for electrode patterns is excluded. The etching process for source/drain electrodes would damage the underlying semiconductor active layer, resulting in degradation of device performance. Ag NPs are considered the primary candidate material to printing electrodes, due to its high conductivity and low temperature treatment in atmospheric ambient [115]. Printed Ag NPs based films have been employed as the source/drain and gate electrodes in some TFTs [115,116,117].

The requirement of printed Ag NPs based electrodes is not only low resistance but also good contact with semiconductor and good morphology. Printed Ag NPs based source/drain electrodes have been commonly reported in p-type organic and carbon-nanotube TFT [118,119,120,121,122,123], though the intrinsic work function of Ag is as low as 4.26 eV [124]. It may be due to the formation of Ag_2_O on a surface that is a high-doped degenerate p-type semiconductor with high work function and moderate electric conductivity. Furthermore, a bottom contact device structure was usually used in these devices, where the source-drain electrodes were printed prior to channel layer. The surface of printed Ag NPs electrodes could be modified to increase work function and improve electrode contact with channel layer, so as to improve device performance [125,126,127,128].

Vacuum-deposited Ag source/drain electrodes have been confirmed very appropriate to contact with n-type oxide semiconductor with a low specific contact resistance, due to its inherent low work function of 4.26 eV [124]. However, for a long time, the oxide thin film transistors were reported, exhibiting low mobility of device (less than 0.5 cm^2^ V^−1^ s^−1^), as printed Ag NPs conductors were utilized as source/drain electrodes [129,130,131]. It suggests that there are some specific issues involving the use of Ag NPs ink in the fabrication process.

Metallic Ag NPs undergo undesirable electrical/thermal migration [115,132], which would result in unpredictable degradation of the device performance [115,122]. Hong et al. reported high-performance oxide thin-film transistors, by surrounding Ag nanoparticles firmly with oleic acid to suppress the migration of Ag inside adjacent oxide semiconductors. The devices exhibited comparable performances to their counterparts of vacuum-deposited metal electrodes. It was also found that the suppressed formation of Ag^2^O and the reduced incorporation of organic components inside the electrodes all play critical roles in facilitating the realization of high performance, oxide thin-film transistors employing printed Ag NPs based source/drain electrodes [115].

Ueoka et al. analyzed printed Ag NPs source/drain electrodes on amorphous indium gallium zinc oxide (IGZO), and found that carbon and hydrogen seriously affect the TFT characteristic. The carbon and hydrogen were abscised from the printed Ag NPs during annealing, generating additional carriers and electron traps [131]. Ning’s group also found carbon at the interface between a-IGZO and printed Ag electrodes. They suggested that the presence of carbon adversely impacted on contact, whereas the diffusion of silver into IGZO semiconductor layer resulted in a better contact at the interface [116]. Recently, the same group reported IGZO TFT with printed Ag source/drain electrodes. The devices show high performance comparable with the analogous devices with sputtered electrodes: a maximum saturation mobility of 8.73 cm^2^ V^−1^ s^−1^ and an average saturation mobility of 6.97 cm^2^ V^−1^ s^−1^, Ion/Ioff ratio more than 107 and subthreshold swing of 0.28 V/decade [133].

To improve electrode contact, the interfaces of printed Ag electrodes with n-type oxide semiconductors were modified. A universal method was reported to produce low-work function electrodes for electronics devices, with surface modifiers based on polymers containing simple aliphatic amine groups [134]. The method was instantiated to achieve high performance solution processed oxide TFTs with inkjet printed Ag source/drain electrodes recently [135]. In other research work, graphene was embedded insert IGZO thin film and printed Ag source-drain electrodes. High-performance IGZO TFTs were achieved with an electron mobility of ~6 cm^2^ V^−1^ s^−1^ and I_on_/I_off_ ratio of ~10^5^ [136].

The morphology of printed Ag line is critical in some cases. More specially, the thickness and profiles of printed Ag line must be well-controlled when it acts as bottom gate electrode in device, to achieve low resistance and low leakage current simultaneously. Usually, a thick film is good for low resistance but results in high leakage current. In addition, smooth surface is required to void electric breakdown. Guo et al. prepared all ink-jet printed low-voltage organic field-effect transistors with Ag NPs based ink, one kind of metal-organic precursor type ink. The printed convex Ag lines have a small thickness of 30 nm and root-mean-square (RMS) roughness of about 1.8 nm [137].

Short channel length of thin film transistors is expected to achieve high operating frequency. There are important technological challenges in printing methods to achieve high-resolution electrodes patterning [111]. Some high-resolution printing equipment has been used to achieve channel length around 1 μm with printed Ag source/drain electrodes, including sub-femtoliter nozzle [138], EHD printer [139], and printing plate with high resolution pattern [140,141]. Recently, Ning’s group reported a short channel length of printed Ag electrodes with a common printer [142,143]. Silver electrodes with 2.4 μm channel length were printed by piezoelectric inkjet printing of 10 pL nozzle, without an extra process. It was attributed to the difference in the retraction velocities on both sides of an ink droplet during the printing process [142].

### 3.3. Biosensors

**Figure 14:**
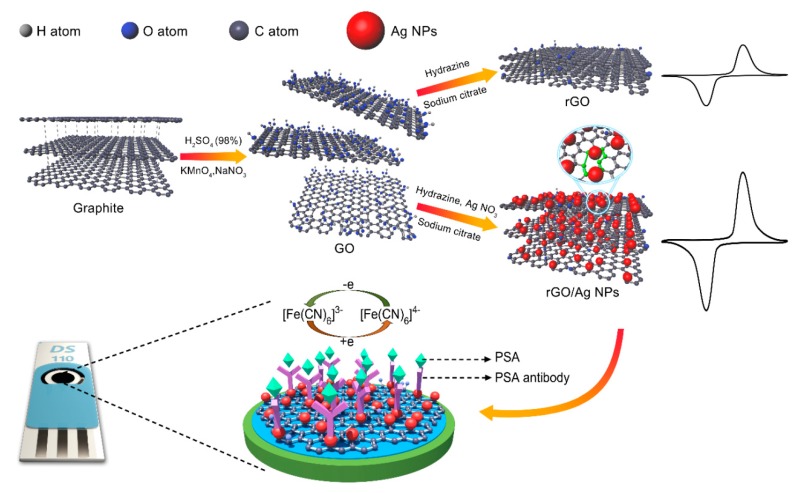
Preparing process of rGO (reduced graphene oxide) and rGO/Ag NPs as well as schematic illustration of fabricated electrochemical immunosensor for PSA (prostate specific antigen) detection. Reproduced with permission from [144]; Copyright 2017 Elsevier.

Ag NPs could be also used as electrode materials for developing electrochemical biosensors. Compared to other metallic NPs, Ag NPs have a relatively low price and superior conductivity, which are undoubtedly the best option for reducing the cost of the biosensors. Han et al. [144] fabricated a label-free electrochemical immunosensor for prostate specific antigen (PSA) detection using rGO/Ag NPs composites as electrode materials on a screen-printed three-electrode system. The rGO/Ag NPs possessed superior electrical conductivity compared to rGO because the small Ag NPs, stabilized by sodium citrate were anchored onto the rGO sheets. The electrochemical immunosensors (EIs) demonstrated a wide linear response range (1.0 to 1000 ng/mL) and low detection limit (0.01 ng/mL). Figure 14 gives the preparing process of rGO and rGO/Ag NPs materials, and the schematic illustration of fabricated electrochemical immunosensor for PSA detection. Ag NPs can speed up the transfer of electrons between the enzyme and the electrode in biosensor, thereby speeding up the reaction and shortening the reaction time. For instance, Rad et al. [145] prepared a hydrogen peroxide sensor having high electron transport efficiency by modifying an electrode with Ag NPs. Wang et al. [146] showed that the gold electrode modified with Ag NPs based nanocomposite demonstrated relatively high sensitivity, fast response time and low detection limit compared to those of their counterpart. In addition, Ag NPs, exhibiting strong localized surface plasmon response (LSPR) absorption in the visible region, have potential as the optical biosensors [147]. Currently printed electrodes for biosensor possess a lot of advantages, such as low price, low sample volume requirement, high sensitivity, high and rapid volume production, portability and easy handling. The Ag NPs based ink supplies a very good opportunity pushing the newly and emerging printed biosensor into the market. It is expected that, in the near future, these printed biosensors will be widely commercialized, and we can use them to find solutions to health monitoring and disease control issues, especially in remote areas.

### 3.4. RFID

RFID (Radio Frequency Identification) tag is a device that provides storing and remote reading of data from items equipped with such tags. The main elements of an RFID tag are a microchip and an antenna that provide power to the tag and are responsible for communication with a reading device. Direct printing of antennas on plastic and paper substrates with the use of Ag NPs inks is a promising approach to the production of RFID tags [148,149,150,151,152,153]. Inkjet printing, screen printing and gravure printing have been used to fabricate Ag NPs based RFID antenna on paper and plastic substrates. The printing parameters, printability of the Ag NPs based ink, properties of the substrates and the sintering methods were investigated to reveal their relationship to the property of RFID tags. Particularly, Jung et al. [151] reported a practical way to provide all-printed and R2R-printable antenna, rectifiers, and ring oscillators on plastic foils and demonstrated 13.56 MHz operated 1-bit RF tags. The whole process used three different printing technologies, R2R gravure, inkjet and pad printing. This is the first report that not only fabricates the antenna but the whole RFID tag by printing. Sanchez-Romaguera et al. [152,153] reported an inkjet and screen printed low cost passive UHF RFID based on Ag NPs, which can be transferred from the tattoo paper to skin. This is significant for the development of wearable electronics and E-skin.

In spite of the printed RFID antenna based on Ag NPs based inks, this is relatively mature in its technique and has shown its obvious advantages in high production efficiency and lower environmental impact; its price is still a little bit high in most cases compared to that of a counterpart fabricated with traditional methods and using aluminum and copper as conductive materials. To overcome this difficulty, the development of conductive inks based on low cost nanometals or carbon is a choice, such as copper, aluminum, nickel, carbon nanotube, graphene, etc. However, such nanometals are easy to oxidize and the attempts to make carbon-based materials into ink and printable have so far not been very successful. On the contrary, to further improve the electric conductivity of the Ag NPs based ink under moderate sintering and sequentially to decrease their dosage in the devices with a relatively thin and uniform printed, coating can be a promising way.

### 3.5. Stretchable Electronics

Fabrication of large-area stretchable electronic devices is necessary for future applications in wearables, healthcare and robotics, etc. For integrating stretchable electronics, stretchable wiring is the most important component. Obtaining reliable conductance against strain could be achieved by the use of intrinsically stretchable materials, such as liquid metals, conducting polymers, and ionic conductors. Another approach is to fabricate conductive pathways using micro-structures, which can be obtained by mainly two methods. One is a metallization of artificially made microstructures, including serpentine, micro- or nano-meshes, or accordion motifs. The other is to develop a conductive nanocomposites mix with conductive fillers and elastomer matrix, which is advantageous in terms of large-area, low-cost and high-throughput fabrication. Among various conductive fillers, including carbon nanotubes, graphene and metallic nanowires, the nano- or micro-Ag with different shapes as well as their composites with other nanomaterials have attracted much attention in recent years.

The use of Ag NPs has yielded a relatively high conductivity at strains larger than 100% [154,155,156,157,158,159]. Matsuhisa et al. [154] reported a high performance stretchable and printable elastic conductor, with the conductivity of 935 S/cm at 400% strain, by the in situ formation of Ag NPs which were created via printing and heating an ink comprising Ag flakes, fluorine rubber, fluorine surfactant and methylisobutylketone (MIBK) as solvents. The results showed that even a small fraction of Ag NPs could reduce the percolation threshold of the composite, increasing the conductivity significantly. Park et al. [155] introduced a conductive and stretchable mat compositing of Ag NPs and rubber fibres. Percolation of the Ag NPs inside the fibres led to a high conductivity of 2200 S/cm at 100% strain for a 150 μm thick mat. Chung et al. [156] introduced a stretchable electrode on wave structured elastomeric substrate by ink jet printed Ag NPs based ink. The printed Ag NPs based electrode showed a relatively good adhesion and conductive stability in the stretching test.

Ag with different shapes and size distributions also have their applications in the stretchable electronics. Ag flakes and fractal structure were used as conductive fillers for their larger contact area compared to that of Ag NPs [160,161,162,163,164,165]. Matsuhisa et al. [160] demonstrated a printable and stretchable conductor that had a conductivity of 182 S/cm and stretchability at a strain of 215% using a nanocomposite material composed of Ag flakes, fluorine rubber and a fluorine surfactant. The fluorine surfactant constitutes a key component that directs the formation of surface-localized conductive networks in the printed elastic conductor, leading to a high conductivity and stretchability. Zhang et al. [165] used fractal structure Ag particles as conductive fillers in PDMS (polydimethylsiloxane) to fabricate a flexible and stretchable conductor, which could stretch up to 100% and twist up to 180° and possessed good mechanical and electronic stability. In addition, a stretchable conductor with Ag nanowires (Ag NWs) embedded in elastomer has been suggested [166,167,168,169,170]. The high aspect ratio of Ag NWs contributed to the high conductivity at a relatively high stain.

## 4. Conclusions

Ag NPs are favored materials for high performance FPE applications because of high electric conductivity, good oxidation resistance and easiness for large-scale preparation. When FPE devices are manufactured, a moderate sintering condition is often required in order to be compatible with heat sensitive substrates and various functional materials. Thus, highly conductive Ag NPs based inks with moderate sintering are essential for FPE applications. The comprehensive research regarding the strategies of achieving highly conductive Ag NPs based ink under moderate sintering has been covered in this review. A better understanding of the relationship of the sintering condition and conductive property of Ag NPs based film with respect to the protective agents, Ag NPs size distribution and shapes, and superficial characterization of the substrates is given. The mechanisms of the various emerging mild and selective sintering technologies are also highlighted. In addition, diverse applications of the Ag NPs based inks and their perspectives in FPE were also presented and discussed, including the transparent conductive film, thin film transistor, biosensor, stretchable electronics and RFID antenna.

Although there has been remarkable progress for Ag NPs based inks in FPE applications, there are still challenges that call for further development in order to gain widely industrial acceptance and in significant quantities. The current high price of the commercial available Ag NPs based inks impedes their wide use for large area flexible and printed electronics. Therefore, research should be focused on the development of new Ag NPs based inks with higher electric conductivity, which could decrease the ink dosage and printed film thickness to achieve the electric performance requirement of the device. For instance, the comprehensive cost of the ultrahigh frequency Ag NPs based RFID antenna fabricated by flexography printing method has already been lower than their counterpart, which uses aluminum as conductive materials and etching technology as the production method. The thickness of the flexography printed RFID antenna is only 0.5–0.8 μm, causing the dosage of Ag NPs to be relatively small and the cost to also be reduced. Meanwhile, for Ag NPs based ink, reduction in sintering temperature could be achieved by lowering the amount of a protective agent or decreasing the bonding energy between Ag and protective agent, which are presented in Section 2.1. However, these processes could adversely affect the stability and printability of the ink, and have unwelcome implications for mechanical integrity and adhesion. Thus, how to make a balance between the electric property, stability and the printability of the Ag NPs based ink is also an urgent problem that needs to be solved. In addition, the underlying physics and chemistry mechanisms governing the sintering process and the interaction between the influence factors of the sintering and electric conductivity in Ag NPs based ink should be further strengthened.

On the other hand, the increasing growth of application in FPE is calling for fast and reliable mild sintering technologies that can perform in high-throughput and R2R manufacturing. The research on emerging sintering techniques presented in this review has created possibilities to a different extent. Among all emerging sintering techniques, IPL sintering is the most promising one. The white-light flashes used in this technique can selectively heat the Ag NPs based ink without damaging the substrate. This sintering technique is also well understood and compatible with the R2R process. In fact, it has already been employed in a pilot production line for fabricating different electrodes and antenna structures. Further strengthening the effort to establish the relationship between different sintering parameters and ink/substrate combination will be likely to push it into the industry as a mainstream technique. One more important perspective in the application of Ag NPs based ink with moderate sintering is 3D printing of conductive patterns. Nowadays, this field is at its very early stages of research and development, and the search for new Ag NPs based ink as well as suitable 3D fabrication tools, is a stimulating challenge for materials scientists. It is likely that a combination of highly conductive Ag NPs based ink with fast and R2R compatible sintering techniques will realize more superior performance of Ag NPs based ink in a wider field of FPE.

## Abbreviations

Ag NPs	Silver nanoparticles
TCF	Transparent conductive film
TFTs	Thin film transistors
FPE	Flexible and printed electronics
PAA	Poly(acrylic acid)
PVP	Poly(vinyl pyrolidone)
PVA	Poly(vinyl alcohol)
DDA	Dodecylamine
DDT	Dodecanethiol
AA	Acetic acid
OA	Oleylamine
CNF	Cellulose nanofibers
EM	Electromagnetic
UV	Ultra-violet
IR	Infra-red
IPL	Intense pulsed light
MOD	Metal organic compounds
NIR	Near-IR
R2R	Roll to roll
RFID	Radio frequency identification
TEM	Transmission electron microscope
PET	Polyethylene terephthalate
rGO	Reduced graphene oxide
PSA	Prostate specific antigen
PDMS	Polydimethylsiloxane
PI	Polyimide
PEN	Polyethylene naphthalate
RES	Rapid electrical sintering
DC	Direct current
AC	Alternating current
ITO	Indium tin oxide
CNTs	Carbon nanotubes
OLEDs	Organic light emitting diodes
IGZO	Indium gallium zinc oxide
RMS	Root-mean-square
PSA	Prostate specific antigen
EIs	Electrochemical immunosensors
LSPR	Localized surface plasmon response
RFID	Radio frequency identification
MIBK	Methylisobutylketone
Ag NWs	Ag nanowires
